# The resurgence of the norovirus GII.4 variant associated with sporadic gastroenteritis in the post-GII.17 period in South China, 2015 to 2017

**DOI:** 10.1186/s12879-019-4331-6

**Published:** 2019-08-06

**Authors:** Liang Xue, Weicheng Cai, Junshan Gao, Le Zhang, Ruimin Dong, Yonglai Li, Haoming Wu, Moutong Chen, Jumei Zhang, Juan Wang, Qingping Wu

**Affiliations:** 1 Guangdong Institute of Microbiology, State Key Laboratory of Applied Microbiology Southern China, Guangdong Provincial Key Laboratory of Microbial Culture Collection and Application, Guangdong Open Laboratory of Applied Microbiology, No. 100, Xianlie Zhong Road, Guangzhou, 510070 People’s Republic of China; 2 Department of Cardiology, Laboratory Department, the Third Affiliated Hospital of Sun Yat-sen University, Guangzhou, 510630 People’s Republic of China; 3 College of Food Science, South China Agricultural University, Guangzhou, 510642 People’s Republic of China

**Keywords:** Norovirus, Acute diarrhea, GII.4, Phylogenetic analyses, Recombination, Evolutionary tracing

## Abstract

**Background:**

Human norovirus is regarded as the leading cause of nonbacterial acute diarrhea in developing and developed countries. Among all genotypes, GII.4 has been the predominant genotype, but in East Asia, it was replaced by the GII.17 in 2014/2015. However, after the prevalence of new GII.17 variant in South China, a sharply increase in the number of norovirus infections associated with sporadic acute diarrhea was detected. In this study, we would investigate the prevalence and genetic diversity of noroviruses in the sporadic acute gastroenteritis cases in the post-GII.17 period in South China.

**Methods:**

Norovirus was screened from 217 patients with sporadic acute gastroenteritis from August 2015 to October 2017 by reverse transcription-polymerase chain reaction. Then, two regions including the partial RNA polymerase and the capsid gene of positive samples were amplified and sequenced. Phylogenetic analyses were performed to determine norovirus genotypes. Complete VP1 sequences of GII.4 strains detected in this study were also amplified and subjected into evolutionary tracing analyses.

**Results:**

A total of 43 (19.82%) norovirus samples were confirmed from 217 stool specimens, and it was found that GII.4 resurged as the new predominant variant, accounting for 76.74% (33/43) of positive samples. Only one local strain GZ2015-L550 was clustered with the contemporary GII.P16/GII.4–2012 recombinant variant, and other 32 local strains belonged to the clade with the GII.Pe/GII.4–2012 variant. Other genotypes including GII.17 (*n* = 4), GII.3 (*n* = 4), GII.8 (*n* = 1) and GI. 6 (n = 1) were also detected. Furthermore, all GII.4 strains were phylogenetic analyzed based on their capsid P2 subdomains. Combined with other reported 754 strains, the GII.4–2012 variant could be divided into two clades. Most GII.4 strains collected in 2016 and 2017 in this study (7/8) formed a new cluster A in Clade II with additional 103 contemporaneous strains. In addition, evolutionary tracing of the capsid P2 subdomain of this variant was also analyzed, and one specific amino acid substitutions (N373) was identified for Cluster A.

**Conclusion:**

In summary, this study confirmed a norovirus infection peak in the post-GII.17 period in South China, which was caused by the resurgence of the GII.4 variant.

**Electronic supplementary material:**

The online version of this article (10.1186/s12879-019-4331-6) contains supplementary material, which is available to authorized users.

## Background

Norovirus (NoVs) is considered to be the main cause of non-bacterial acute gastroenteritis in developed and developing countries. This virus infects people of all ages and is responsible for almost one-fifth of all acute gastroenteritis cases worldwide [1]. This pathogen is responsible for almost half of all foodborne gastroenteritis outbreaks, and it is estimated that every year 200,000 children under 5 years old die from NoV associated gastroenteritis in developing countries [2, 3]. Huge medical economic and social losses caused by this virus have been estimated [4]. This virus can be transmitted by the fecal-oral pathway through contaminated environments, food, water, and person-to-person contact. Extensive NoV outbreaks are more prominent in semi-enclosed areas, including school, hospitals, nursing homes, cruise ships and holiday resorts [5]. NoV vaccine candidates have been tested in phase 1 and phase 2 clinical trials [6, 7], but there are still no licensed virus vaccines or effective medicine treatments against NoV infections as of this writing.

As a member of the family *Caliciviridae*, NoV is a single strand, positive-sense RNA virus. Its genome is approximately 7.5 kb to 7.7 kb in size and contains three open reading frames (ORFs) [8]. Given its high genetic diversity, NoVs could be classified into seven genogroups (GI to GVII) based on the complete major capsid protein VP1 sequences [9, 10]. GI and GII NoVs are the genogroups primarily responsible for humans, and GIV could also infect humans. Genogroups could be further divided into more than 40 genotypes, and GI and GII include at least 9 and 22 genotypes, respectively [11]. Of all genotypes, GII.4 has been identified as the most predominant genotype for 70~80% of NoV outbreaks in most countries over the past 20 years [12]. The global pandemic spread of the GII.4 variant (US95/96) was first recognized in the mid-1990s. Then, after the emergence of GII.4 Farmington Hills 2002, new GII.4 variants appeared every 2 to 3 years and could spread globally within months [13]. In addition, a serial of non-GII.4 epidemic variants emerged recently have drawn extensive attention, especially the GII.17 Kawasaki 2014 in the winter of 2014/2015 [14–16].

In China, NoV gastroenteritis is also a public health concern, and this virus has been identified as one of the first two causes of acute diarrhea for more than a decade [17, 18]. A series of globally prevalent GII.4 variants have also been found in China, along with the GII.17 Kawasaki 2014 variant. However, a recent study showed that GII.17 did not completely replace GII.4, and a GII.P16/GII.4–2012 recombinant was detected as a new predominant variant [19, 20]. Recently, we detected a sharply increase in the number of NoV infections associated with sporadic acute diarrhea in South China. Increasing NoV infection activity is generally associated with the emergence of new epidemic variants. To give early warning of the next upcoming NoV season, we conducted a pilot study to characterize the prevalence and evolution patterns of NoV variants in the post-GII.17 period.

## Methods

### Fecal specimen collection

From August 2015 to October 2017, 217 fecal specimens were collected from patients suffering from acute diarrhea at the Third Affiliated Hospital of Sun Yat-sen University in Guangzhou, South China. All specimens were stored at − 80 °C until further analyses.

### Sample treatment, RNA extraction and NoV detection

A 10% (w/v) stool suspension was first prepared using phosphate-buffered saline (diethylpyrocarbonate-treated) and centrifuged at 10000 g for 1 min at 4 °C. Viral RNA was then extracted from 140 μL of the supernatant using a QIAamp Viral RNA Mini Kit (QIAGEN, Hilden, Germany) according to the manufacturer’s instructions. One-step reverse transcription-polymerase chain reaction (RT-PCR) was employed for NoV detection using a one-step RT-PCR kit (TAKARA, Dalian, China) in this study. Genogroup-specific primers of G1SKF/G1SKR and G2SKF/G2SKR were used to screen for GI and GII NoV strains by amplifying the capsid N/S domain (region C in ORF2) [21]. Primers JV12Y and JV13I were also chosen to detect NoVs by amplifying a partial RNA-dependent RNA polymerase (RdRp) region (region A in ORF1) [22]. Besides, the primer set of NV2of2 and GV132 was used to amplify the full-length GII.4 NoV VP1 gene [23]. All tests were conducted in different rooms to avoid cross-contamination. In each run, RNase-Free distilled water served as a negative control, and a NoV-positive stool sample was used as a positive control.

### Sequencing and phylogenetic analyses

After analyzed agarose gel electrophoresis, positive amplicons were purified from agarose gels using DNA extraction kits (Magentec, Guangzhou, China) following the manufacturer’s instructions, and then were subjected to direct sequencing using amplification primers. DNA sequencing was performed in the ABI Prism 3730XL Genetic Analyzer (Applied Biosystems, Foster City,CA, USA) using the BigDye® Terminator v3.1 Cycle Sequencing Kit, which was carried out by Majorbio Co., Ltd. (Shanghai, China). Nucleotide sequences data of detected NoV strains in this study were deposited in GenBank under accession numbers MH469166-MH469208.

All nucleotide sequences were first edited with the BioEdit® Sequence Alignment Editor software (v.7.0.1). NoV nucleotide sequences or derived amino acid sequences detected in this study with reference sequences of different genotypes were aligned using the ClustalX algorithm v1.83 with the default parameters [24]. For further phylogenetic analysis of local GII.4 NoV strains during the period from August 2015 to October 2017, a total of 754 reference GII.4 strains (including 751 GII.4–2012 strains, one GII.4–2009 strain, one GII.4-2006b strain, and one GII.4–2004) were also selected, and their information was listed in the Additional file 1 Table S2. We inferred maximum likelihood trees with Molecular Evolutionary Genetics Analysis (MEGA) v7.0 software [25], and best substitution models for the dataset were chosen based with the lowest Bayesian Information Criterion scores. Besides, neighbor–joining trees were also conducted with MEGA v7.0 software using the Kimura two-parameter model for nucleotide sequences. The reliability of the phylogenetic tree was assessed by bootstrap sampling of 1000 replicates. All nucleotide sequences were also submitted to the online NoV Genotyping Tool (version 2.0) (https://www.rivm.nl/mpf/typingtool/norovirus/) to verify phylogenetic results [26].

## Results

### Prevalence of NoVs associated with sporadic acute diarrhea in South China

A total of 217 fecal specimens were collected from patients with acute diarrhea at the sentinel hospital from August 2015 to October 2017. Using the primer set targeting the capsid N/S domain, 43 samples (19.82%) were detected NoV positive, 42 of which were identified by G2SKF/G2SKR primers, and only one identified by G1SKF/G1SKR primers (Table 1).

**Table 1:** Positive rates and distribution of NoVs genotypes detected in South China, from 2015 to 2017

Year	Samples collected	Positive samples	Positive samples of different genotypes				
			GI.6	GII.3	GII.4	GII.8	GII.17
2015	61	5 (8.20%)			4 (6.56%)		1
2016	91	26 (28.57%)	1	4	19 (20.88%)		2
2017	65	12 (18.46%)			10 (15.38%)	1	1
Total	217	43 (19.82%)	1	4	33 (15.21%)	1	4

Among all, 93 fecal samples were collected from female patients with the NoV positive rate of 21.51%, which was a little higher than that of male patients (23/124, 18.55%). Fifteen NoV positive samples were collected from children under 5 years old (*n* = 56) with the NoV positive rate of 26.79%, and three positive samples were collected from older children and teenagers between 5 and 18 years old (*n* = 11) with the positive rate of 27.27%, which were both higher than that of the adult group (28/161, 17.39%) (Table 2).

**Table 2:** Demography of NoV-positive patients detected in this study

Patient profile	Number of samples collected	Number of positive sample (%)
Gender		
Male	124	23 (18.55%)
Female	93	20 (21.51%)
Age		
<5 years old	56	15 (26.79%)
5–18 years old	11	3 (27.27%)
>18 years old	150	25 (16.67%)

### Genetic diversity of NoV strains during this surveillance

**Fig. 1:**
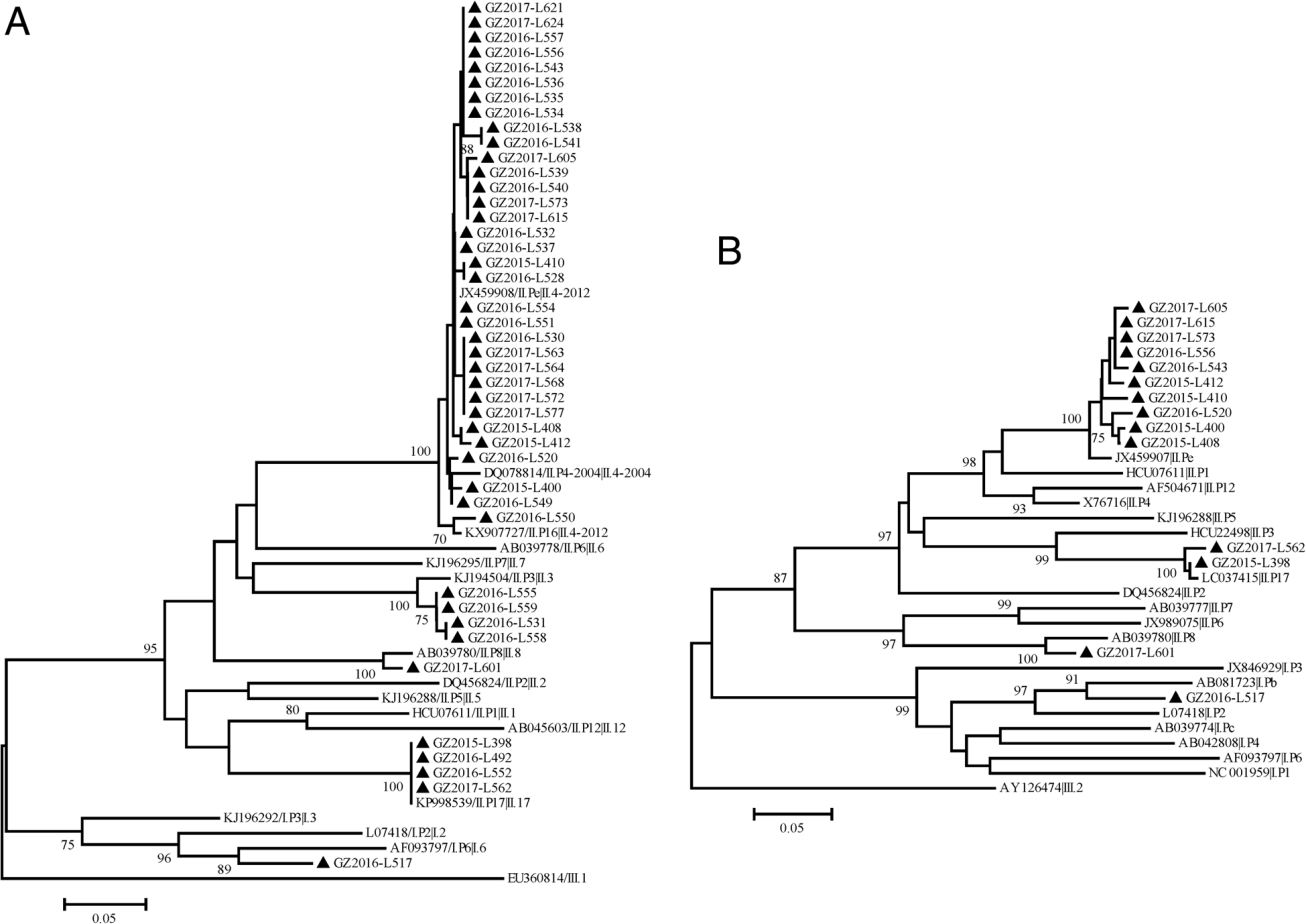
Phylogenetic trees of NoV strains based on the nucleotide sequences of (**a**) the partial capsid gene and (**b**) the partial RNA-dependent RNA polymerase gene. The dendrogram was constructed by the neighbor-joining method with the Kimura two-parameter model in MEGA v7.0 [25]. Numbers at the nodes indicate supporting bootstrap values (%) for 1000 resampled datasets; only values greater than 70% are shown. The scale bar represents the unit for the expected number of substitutions per site. Local NoV strains from this study are labelled by black triangles

**Fig. 2:**
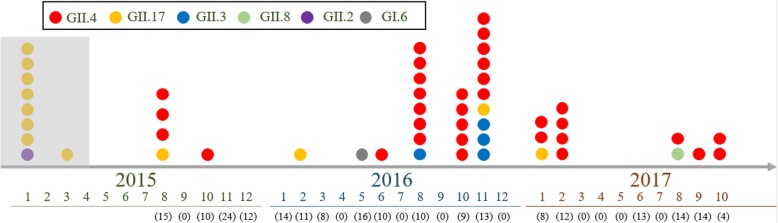
Temporal distribution of norovirus positive strains detected during the period from August 2015 to October 2017, in South China. Norovirus genotypes are represented in this study by different colors in the legend (GII.4, red; GII.17, yellow; GII.3, blue; GII.8, green; GI.6, grey). And the numbers of samples tested per month were listed below the collecting time, which were in parentheses

To identify the genotypes of the detected NoVs strains, nucleotide sequences of the capsid N/S domain (*n* = 43) amplified by G1SKF/G1SKR or G2SKF/G2SKR and the partial RdRp region (*n* = 14) amplified by JV12Y/JV13I were obtained for phylogenetic analyses. Only one strain, which collected from a seven-month-old baby boy in May 2016, belonged to GI genogroup (GI.Pb/GI.6). Other 42 strains were clustered as GII genogroup, consisting of four genotypes (Fig. 1a). Most of these strains belonged to GII.4 (*n* = 33, 76.74%), and among them, partial RdRp regions from ten GII.4 strains were amplified and identified as GII. Pe genotype. The positive rate of GII.4 NoVs reached 20.88% (2016) during the monitoring. Especially between August 2016 and February 2017, 24 GII.4 strains were detected with the positive rate of 46.15% (Fig. 2). In addition, four GII.17 positive samples were detected every year during the study period (August 2015, February and November 2016, January 2017), and two of them were also verified by the partial RdRp regions as GII.P17. Four GII.3 positive samples were also detected, which collected in August and November 2016. And one GII.P8/GII.8 strain was detected from a 27-year-old female patient in August 2017, which was the first one detected in mainland China.

### Phylogenetic analyses and evolutionary tracing of GII.4–2012 strains based on the capsid P2 subdomain

**Fig. 3:**
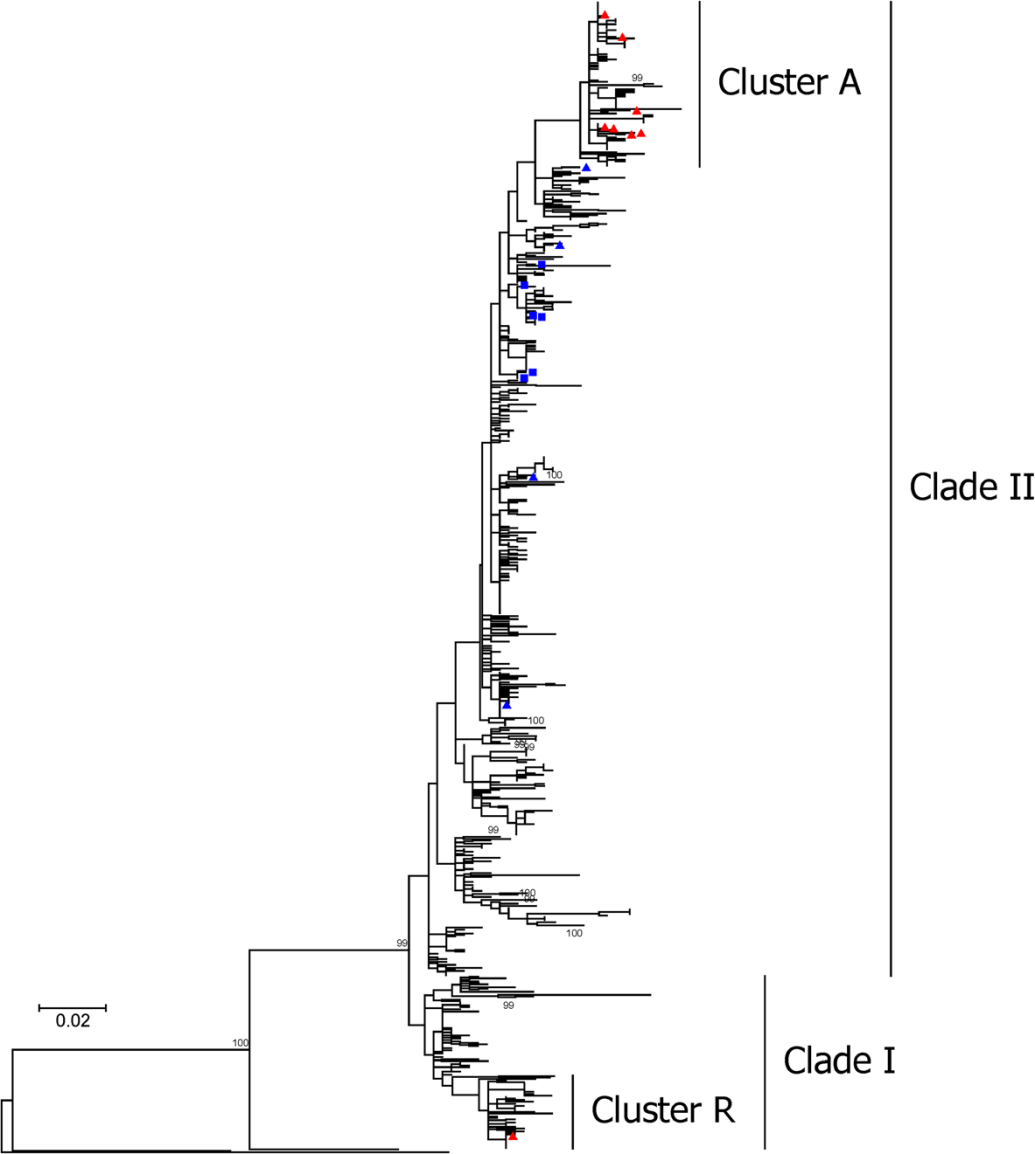
Phylogenetic analysis of GII.4 NoV strains detected in South China during the period from August 2015 to October 2017, based on the nucleotide sequences of the capsid P2 subdomain. Phylogeny was reconstructed using the maximum-likelihood method implemented in MEGA v7.0 [25] with the TN93 + G + I model (best nucleotide substitution model for the dataset based with the lowest Bayesian Information Criterion scores). Numbers at the nodes indicate supporting bootstrap values (%) for 1000 resampled datasets, and bootstrap values of selected branches are shown (> 70%). The scale bar represents the unit for the expected number of substitutions per site. Local NoV strains from this study are labelled by red triangles (for strains in 2016 and 2017) and blue triangles (for strains in 2014 and 2015), respectively. Local NoV strains from previous studies are labelled by blue squares. Information of all reference sequences used for phylogenetic analysis in this figure was listed in the Additional file 1 Table S2

For further understanding the cause of the sharply increase in the prevalence of NoV infections, local GII.4 strains (12 in this study, 6 in our previous study) were phylogenetically analyzed with other reference GII.4 strains (751 GII.4–2012 strains, one GII.4–2009 strain, one GII.4-2006b strain, and one GII.4–2004). Inferred ML trees based on their capsid P2 subdomains showed that all GII.4–2012 strains (*n* = 766) could be divided into two clades I (*n* = 115) and II (*n* = 651) (Fig. 3). Most local GII.4 strains collected in 2016 and 2017 in this study (7/8) formed a new cluster A in Clade II with other contemporaneous GII.4–2012 strains (*n* = 103). And one local strain GZ2016-L550 was clustered with other GII.P16/GII.4–2012 strains (*n* = 48) as Cluster R in Clade I.

**Fig. 4:**
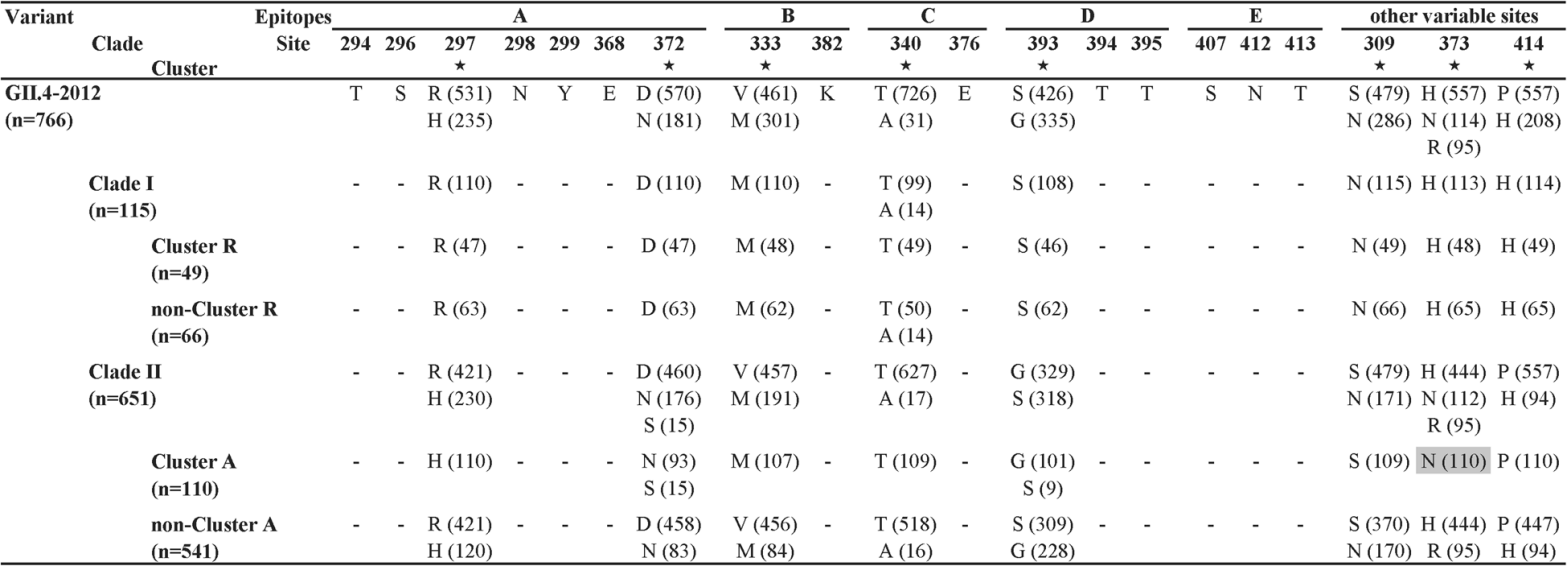
Evolutionary tracing of variable sites on the capsid P2 subdomain of GII.4–2012 strains. All variable sites are marked by asterisks below. Conserved amino acid sites in five epitopes A-E were only listed for the GII.4–2012 variant, and then replaced by dashes “-” for different clades and clusters. The specific amino acid substitution N373 for Cluster A is highlighted in light gray

Based on multi-alignment results, evolutionary tracing of above GII.4–2012 strains were subsequently performed, mainly focusing on five epitopes (A-E) and other three variable sites in the P2 subdomain (Fig. 4). The variable site was defined as being conserved in less than 95% of all strains. Only eight variable sites were identified for this variant, five of which located in the epitopes (A-D). For strains in Cluster R, their amino acid substitutions at these variable sites were consistent with those of other strains in Clade I. However, for strains in Cluster A, their amino acid substitutions at these variable sites were not completely consistent with those of other strains in Clade II, especially N373 only specific to Cluster A.

## Discussion

After the emergence of GII.17 Kawasaki 2014 in South China, a sharply increase of NoV infections was found from August 2015 to October 2017. NoV positive rate was almost two times higher than those in our previous studies (Additional file 1 Table S1) [17, 18]. Findings in this study suggested that the resurgence of the GII.4 variant caused increased levels of NoV activity in the post GII.17 period. More interestingly, the GII.4 strains appeared in 2016 and 2017 had an obvious phylogenetic difference with previous ones.

NoVs are regarded as the primary cause of acute viral gastroenteritis worldwide [3]. Despite its broad genetic diversity, GII.4 has been the predominant genotype in outbreaks and sporadic infections since its global pandemic [12]. The emergence of novel GII.4 epidemic variants has been continuously updated until the replacement of GII.4 Sydney 2012 by the novel GII.17 Kawasaki 2014 in 2014/2015 [15, 27]. It was of great significance for the pandemic of GII.17 Kawasaki 2014 as the first non-GII.4 epidemic variant, and it was even suggested whether GII.17 be the end of the GII.4 era [28]. However, epidemiological surveillance data showed that GII.17 was detected less frequently after 2015 [27].

NoV epidemic and evolutionary characteristics after the emergence and prevalence of GII.17 is an important issue. Surveillance data from the NoroNet network suggested that no GII.4 drift variants emerged after 2012, and instead, the GII.42012 capsid seemed to persist through recombination. A novel GII.P16/GII.4–2012 variant has been detected in some countries since 2014, including China [19, 20]. In our study, only one strain GZ2016-L550 was detected as this recombinant genotype. Therefore, it was not considered to be the main reason for the increase in sporadic NoV infections in South China. Based on VP1 sequences, GII.P16/GII.4–2012 strains were phylogenetically separated from previously circulating GII.4–2012 strains, but their antigenic domains in the capsid remains unchanged [29]. Besides, two other recombinants of GII.Pe/GII.4–2012 and GII.P4–2009/GII.4–2012 were also reported co-circulating with the GII.P16/GII.4–2012 [27, 30]. In addition to antigenic evolution, recombination is an important evolutionary pattern for GII.4 genotype, and three recombination hot spots have been reported, including near the ORF1/ORF2 and ORF2/ORF3 overlaps, as well as within ORF2 [31]. However, the effect of GII.4 recombination on its epidemic should be verified in the next work.

More importantly, the GII.4 strains in the new Cluster A, which evolved by amino acid substitutions, were predicted as the cause of the sharply increase of NoV infections in this study. When compared with the previous GII.4 strains, some obvious specific amino acid substitutions were identified (especially N373) [17]. It has been reported that under the pressure of herd immunity, the antigenicity of capsid protein VP1 gradually alters through variants selection [32, 33]. During the epidemic of the GII.4–2012 variant, most of the variable amino acid sites (5/8) located in the previously reported epitopes (297, 372 in Epitope A, 333 in Epitope B, 340 in Epitope C, 393 in Epitope D). The change of one amino acid in the key epitope could cause the effect of virus immunogenicity, so the significance of these amino acid substitutions should be determined using antibody experiments in future work [13]. However, it should be noted that the bootstrap support on the root node of clade A is not more than 70 (only 48). Therefore, the novelty of N373 to clade A is supported by the inferred ML tree (Fig. 3), but N373 may not be specific to a single cluster.

We reported a new Cluster A consisted by recently detected GII.4–2012 strains in the post-GII.17 period, which was predicted to be responsible for the sharply increase in NoV prevalence in South China. This result was not completely consistent with the report based on surveillance data from the NoroNet network [27]. This inconsistency was mainly due to the different collection times of isolated strains. In addition, the limitation of our study was that our results were not from nationwide surveillance. However, newly emerged GII.4 variants could spread globally within months after its emergence [13], and the similar situations for GII.4–2012 and GII.17 Kawasaki 2014 were also reported in China [34].

## Conclusion

In conclusion, the resurgence of the GII.4 variant in the post-GII.17 period caused a sharply increase in the number of sporadic infection cases in South China. Most local GII.4 strains evolved by amino acid substitutions and clustered into a new GII.4–2012 Cluster A. Meanwhile, the recently reported GII.4–2012 recombinant, which mainly caused outbreaks, was also rarely detected in this sporadic infection surveillance.

## Additional file

Additional file 1: **Table S1**. Comparison of different studies about NoV prevalence associated with sporadic gastroenteritis in Guangzhou. **Table S2**. Information of norovirus reference sequences used in this study. (DOCX 103 kb)

## Data Availability

All data generated or analyzed during this study are included in this published article. The sequences of the NoV strains obtained in this study were deposited in the GenBank under the accession numbers MH469166-MH469208.

## Abbreviations

GI: Genogroup I
GII: Genogroup II
MEGA: Molecular evolutionary genetic analysis
NoV: Norovirus
ORF: Open reading frames
RdRp: RNA-dependent RNA polymerase
RT-PCR: Reverse transcription polymerase chain reaction
