# Supplementary material for: The resurgence of the norovirus GII.4 variant associated with sporadic gastroenteritis in the post-GII.17 period in South China, 2015 to 2017
Source: BMC Infect Dis. 2019 Aug 6;19:696. doi: 10.1186/s12879-019-4331-6 (PMC6683363; doi:10.1186/s12879-019-4331-6)
Supplement: Supplementary file 1 — Table S1. Comparison of different studies about NoV prevalence associated with sporadic gastroenteritis in Guangzhou. Table S2. Information of norovirus reference sequences used in this study. (DOCX 103 kb) [file 12879_2019_4331_MOESM1_ESM.docx]

Table S1 Comparison of different studies about NoV prevalence associated with sporadic gastroenteritis in Guangzhou

| Reference | Time Span | Number of samples collected | Number of positive samples | Positive rate |
| --- | --- | --- | --- | --- |
| [18] | 2010.11-2011.1 | 89 | 9 | 10.11% |
| [17] | 2013.11-2014.3  2014.11-2015.3 | 215 | 25 | 11.63% |
| This study | 2015.8-2017.10 | 217 | 43 | 19.82% |

**References**

17. Xue L, Dong RM, Wu QP, Li YL, Cai WC, Kou XX, Zhang JM, Guo WP: Molecular epidemiology of noroviruses associated with sporadic gastroenteritis in Guangzhou, China, 2013-2015. Arch Virol. 2016; 161(5):1377-84.

18. Xue L, Wu QP, Dong RM, Kou XX, Li YL, Zhang JM, Guo WP: Genetic analysis of noroviruses associated with sporadic gastroenteritis during winter in Guangzhou, China. Foodborne Pathog Dis. 2013; 10(10):888-95.

Table S2 Information of norovirus reference sequences used in this study

| GenBank Accession No. | Year | Country | RdRp-Genotype |
| --- | --- | --- | --- |
| AB972466 | 2012 | JP | GII.Pe |
| AB972467 | 2012 | JP | GII.Pe |
| AB972468 | 2012 | JP | GII.Pe |
| AB972469 | 2012 | JP | GII.Pe |
| AB972470 | 2012 | JP | GII.Pe |
| AB972472 | 2012 | JP | GII.Pe |
| AB972473 | 2012 | JP | GII.Pe |
| AB972475 | 2012 | JP | GII.Pe |
| AB972477 | 2012 | JP | GII.Pe |
| AB972478 | 2012 | JP | GII.Pe |
| AB972479 | 2012 | JP | GII.Pe |
| AB972480 | 2012 | JP | GII.Pe |
| AB972481 | 2012 | JP | GII.Pe |
| AB972482 | 2012 | JP | GII.Pe |
| AB972483 | 2012 | JP | GII.Pe |
| AB972484 | 2012 | JP | GII.Pe |
| AB972485 | 2012 | JP | GII.Pe |
| AB972487 | 2012 | JP | GII.Pe |
| AB972488 | 2012 | JP | GII.Pe |
| AB972489 | 2012 | JP | GII.Pe |
| AB972490 | 2012 | JP | GII.Pe |
| AB972491 | 2012 | JP | GII.Pe |
| AB972492 | 2012 | JP | GII.Pe |
| AB972493 | 2012 | JP | GII.Pe |
| AB972494 | 2012 | JP | GII.Pe |
| AB972496 | 2012 | JP | GII.Pe |
| AB972497 | 2012 | JP | GII.Pe |
| AB972498 | 2012 | JP | GII.Pe |
| AB972499 | 2011 | JP | GII.Pe |
| AB972502 | 2011 | JP | GII.Pe |
| AB972503 | 2011 | JP | GII.Pe |
| AB972504 | 2011 | JP | GII.Pe |
| AB972505 | 2011 | JP | GII.Pe |
| AB972507 | 2012 | JP | unknown |
| AB972508 | 2012 | JP | unknown |
| AB972510 | 2012 | JP | unknown |
| AB972512 | 2012 | JP | unknown |
| AB972516 | 2012 | JP | unknown |
| AB972517 | 2012 | JP | unknown |
| AB972518 | 2012 | JP | unknown |
| AB972519 | 2012 | JP | unknown |
| AB972520 | 2012 | JP | unknown |
| AB972521 | 2012 | JP | unknown |
| AB972522 | 2012 | JP | unknown |
| AB972523 | 2012 | JP | unknown |
| AB972524 | 2012 | JP | unknown |
| AB972527 | 2012 | JP | unknown |
| AB972529 | 2012 | JP | unknown |
| AB972530 | 2012 | JP | unknown |
| AB972531 | 2012 | JP | unknown |
| AB972532 | 2012 | JP | unknown |
| AB972533 | 2012 | JP | unknown |
| DQ078814 | 2004 | AU | GII.P4\|GII.4-2004 |
| GU445325 | 2009 | US | GII.Pe |
| JQ911598 | 2009 | VNM | GII.P4\|GII.4-2006b |
| JX459907 | 2012 | AU | GII.Pe |
| JX629458 | 2012 | CUHK | unknown |
| KC175323 | 2012 | CN | GII.Pe |
| KC243078 | 2012 | CNTW | unknown |
| KC456072 | 2012 | CN | unknown |
| KC473544 | 2012 | CN | unknown |
| KC473546 | 2012 | CN | unknown |
| KC517361 | 2012 | CNTW | GII.Pe |
| KC517362 | 2012 | CNTW | GII.Pe |
| KC517364 | 2012 | CNTW | GII.Pe |
| KC517370 | 2012 | CNTW | unknown |
| KC577175 | 2012 | CN | GII.Pe |
| KC631827 | 2012 | CN | GII.Pe |
| KC695807 | 2012 | AU | unknown |
| KC792280 | 2012 | CNTW | unknown |
| KF008243 | 2012 | FR | unknown |
| KF008244 | 2012 | FR | unknown |
| KF059999 | 2012 | AU | unknown |
| KF060004 | 2012 | AU | unknown |
| KF060009 | 2012 | AU | unknown |
| KF060021 | 2012 | AU | unknown |
| KF060023 | 2012 | AU | unknown |
| KF060026 | 2012 | AU | unknown |
| KF060029 | 2012 | AU | unknown |
| KF060030 | 2012 | AU | unknown |
| KF060031 | 2012 | AU | unknown |
| KF060041 | 2012 | AU | unknown |
| KF060056 | 2012 | AU | unknown |
| KF060062 | 2012 | AU | unknown |
| KF060073 | 2012 | AU | unknown |
| KF060076 | 2012 | AU | unknown |
| KF060080 | 2012 | AU | unknown |
| KF060087 | 2012 | AU | unknown |
| KF060089 | 2012 | AU | unknown |
| KF060096 | 2012 | AU | unknown |
| KF060097 | 2012 | AU | unknown |
| KF060102 | 2012 | AU | unknown |
| KF060110 | 2012 | AU | unknown |
| KF060111 | 2012 | AU | unknown |
| KF060112 | 2012 | AU | unknown |
| KF060113 | 2012 | AU | unknown |
| KF060125 | 2012 | NZ | unknown |
| KF060126 | 2012 | NZ | unknown |
| KF060127 | 2012 | NZ | unknown |
| KF060129 | 2012 | NZ | unknown |
| KF060130 | 2012 | NZ | unknown |
| KF060131 | 2012 | NZ | unknown |
| KF060132 | 2012 | NZ | unknown |
| KF060133 | 2012 | NZ | unknown |
| KF060134 | 2012 | NZ | unknown |
| KF060135 | 2012 | NZ | unknown |
| KF060136 | 2012 | NZ | unknown |
| KF060137 | 2012 | NZ | unknown |
| KF060138 | 2012 | NZ | unknown |
| KF060139 | 2012 | NZ | unknown |
| KF060140 | 2012 | NZ | unknown |
| KF060141 | 2012 | NZ | unknown |
| KF060142 | 2012 | NZ | unknown |
| KF060143 | 2012 | NZ | unknown |
| KF060144 | 2012 | NZ | unknown |
| KF060145 | 2012 | NZ | unknown |
| KF060146 | 2012 | NZ | unknown |
| KF060147 | 2012 | NZ | unknown |
| KF060148 | 2012 | NZ | unknown |
| KF060149 | 2012 | NZ | unknown |
| KF060150 | 2012 | NZ | unknown |
| KF060151 | 2012 | NZ | unknown |
| KF060152 | 2012 | NZ | unknown |
| KF060153 | 2012 | NZ | unknown |
| KF145148 | 2012 | JP | GII.Pe |
| KF145149 | 2012 | JP | GII.Pe |
| KF177429 | 2012 | AU | unknown |
| KF177430 | 2012 | AU | unknown |
| KF177431 | 2012 | AU | unknown |
| KF177432 | 2013 | AU | unknown |
| KF177433 | 2012 | AU | unknown |
| KF177434 | 2013 | AU | unknown |
| KF177435 | 2013 | AU | unknown |
| KF177436 | 2013 | AU | unknown |
| KF177437 | 2013 | AU | unknown |
| KF177438 | 2013 | AU | unknown |
| KF177439 | 2012 | AU | unknown |
| KF177440 | 2012 | AU | unknown |
| KF177441 | 2012 | AU | unknown |
| KF177442 | 2012 | AU | unknown |
| KF177443 | 2012 | AU | unknown |
| KF177444 | 2013 | AU | unknown |
| KF177445 | 2012 | AU | unknown |
| KF177446 | 2012 | AU | unknown |
| KF177447 | 2012 | AU | unknown |
| KF177448 | 2012 | AU | unknown |
| KF177449 | 2012 | AU | unknown |
| KF306214 | 2013 | CN | GII.Pe |
| KF378731 | 2013 | IT | GII.P4 |
| KF509946 | 2012 | CAN | GII.Pe |
| KF668569 | 2012 | IT | GII.Pe |
| KJ196279 | 2012 | JP | GII.Pe |
| KJ196280 | 2012 | JP | GII.Pe |
| KJ196281 | 2012 | JP | GII.Pe |
| KJ196293 | 2012 | JP | GII.Pe |
| KJ196296 | 2012 | CNTW | GII.Pe |
| KJ433968 | 2012 | TW | unknown |
| KJ433969 | 2012 | TW | unknown |
| KJ433970 | 2012 | TW | unknown |
| KJ451059 | 2012 | TW | GII.Pe |
| KJ451060 | 2012 | TW | GII.Pe |
| KJ533132 | 2012 | TW | GII.Pe |
| KJ533134 | 2012 | TW | GII.Pe |
| KJ649701 | 2014 | CUHK | unknown |
| KJ649702 | 2014 | CUHK | unknown |
| KJ649703 | 2014 | CUHK | unknown |
| KJ649704 | 2014 | CUHK | unknown |
| KJ649705 | 2013 | CUHK | GII.Pe |
| KJ678134 | 2012 | CN | unknown |
| KJ678135 | 2012 | CN | unknown |
| KJ678136 | 2012 | CN | unknown |
| KJ678137 | 2013 | CN | unknown |
| KJ678138 | 2013 | CN | unknown |
| KJ678139 | 2013 | CN | unknown |
| KJ678140 | 2013 | CN | unknown |
| KJ678141 | 2013 | CN | unknown |
| KJ678142 | 2013 | CN | unknown |
| KJ678143 | 2013 | CN | unknown |
| KJ678144 | 2013 | CN | unknown |
| KJ678145 | 2013 | CN | unknown |
| KJ678146 | 2013 | CN | unknown |
| KJ678147 | 2013 | CN | unknown |
| KJ678148 | 2013 | CN | unknown |
| KJ678149 | 2013 | CN | unknown |
| KJ678150 | 2013 | CN | unknown |
| KJ678151 | 2013 | CN | unknown |
| KJ678152 | 2013 | CN | unknown |
| KJ678153 | 2013 | CN | unknown |
| KJ678154 | 2013 | CN | unknown |
| KJ685406 | 2012 | BGD | GII.Pe |
| KJ685412 | 2012 | BGD | GII.Pe |
| KJ710247 | 2012 | ZA | GII.Pe |
| KJ716356 | 2012 | CN | unknown |
| KJ716358 | 2012 | CN | unknown |
| KJ716359 | 2012 | CN | unknown |
| KJ716360 | 2012 | CN | unknown |
| KJ716363 | 2013 | CN | unknown |
| KJ716365 | 2013 | CN | unknown |
| KJ716366 | 2013 | CN | unknown |
| KJ716367 | 2013 | CN | unknown |
| KJ716368 | 2013 | CN | unknown |
| KJ735099 | 2011 | MOR | unknown |
| KJ955492 | 2012 | CN | GII.Pe |
| KM114291 | 2014 | CN | unknown |
| KM245075 | 2013 | TW | GII.Pe |
| KM258128 | 2012 | TW | GII.Pe |
| KM268076 | 2014 | CUHK | unknown |
| KM268077 | 2014 | CUHK | unknown |
| KM268078 | 2014 | CUHK | unknown |
| KM268079 | 2014 | CUHK | unknown |
| KM268080 | 2014 | CUHK | unknown |
| KM268081 | 2014 | CUHK | unknown |
| KM268082 | 2014 | CUHK | unknown |
| KM268083 | 2014 | CUHK | unknown |
| KM268084 | 2014 | CUHK | unknown |
| KM268085 | 2014 | CUHK | unknown |
| KM268086 | 2014 | CUHK | unknown |
| KM268087 | 2014 | CUHK | unknown |
| KM268088 | 2014 | CUHK | unknown |
| KM268089 | 2014 | CUHK | unknown |
| KM268090 | 2014 | CUHK | unknown |
| KM268091 | 2014 | CUHK | unknown |
| KM268092 | 2014 | CUHK | unknown |
| KM268093 | 2014 | CUHK | unknown |
| KM268094 | 2014 | CUHK | unknown |
| KM268095 | 2014 | CUHK | unknown |
| KM268096 | 2014 | CUHK | unknown |
| KM268097 | 2014 | CUHK | unknown |
| KM268098 | 2014 | CUHK | unknown |
| KM268099 | 2014 | CUHK | unknown |
| KM268100 | 2014 | CUHK | unknown |
| KM268101 | 2014 | CUHK | unknown |
| KM268102 | 2014 | CUHK | unknown |
| KM268103 | 2014 | CUHK | unknown |
| KM268104 | 2014 | CUHK | unknown |
| KM268105 | 2014 | CUHK | unknown |
| KM268106 | 2014 | CUHK | unknown |
| KM268107 | 2014 | CUHK | unknown |
| KM514057 | 2014 | CUHK | unknown |
| KM514058 | 2014 | CUHK | unknown |
| KM514059 | 2014 | CUHK | unknown |
| KM514060 | 2014 | CUHK | unknown |
| KM514061 | 2014 | CUHK | unknown |
| KM514062 | 2014 | CUHK | unknown |
| KM514063 | 2014 | CUHK | unknown |
| KM514064 | 2014 | CUHK | unknown |
| KM514065 | 2014 | CUHK | unknown |
| KM514066 | 2014 | CUHK | unknown |
| KM514067 | 2014 | CUHK | unknown |
| KM514069 | 2014 | CUHK | unknown |
| KM514070 | 2014 | CUHK | unknown |
| KM514072 | 2014 | CUHK | unknown |
| KM514073 | 2014 | CUHK | unknown |
| KM514074 | 2014 | CUHK | unknown |
| KM514075 | 2014 | CUHK | unknown |
| KM514076 | 2014 | CUHK | unknown |
| KM514077 | 2014 | CUHK | unknown |
| KM514078 | 2014 | CUHK | unknown |
| KM514079 | 2014 | CUHK | unknown |
| KM982935 | 2014 | CUHK | unknown |
| KM982936 | 2014 | CUHK | unknown |
| KM982937 | 2014 | CUHK | unknown |
| KM982938 | 2014 | CUHK | unknown |
| KM982939 | 2014 | CUHK | unknown |
| KM982940 | 2014 | CUHK | unknown |
| KM982941 | 2014 | CUHK | unknown |
| KM982942 | 2014 | CUHK | unknown |
| KM982943 | 2014 | CUHK | unknown |
| KM982944 | 2014 | CUHK | unknown |
| KM982945 | 2014 | CUHK | unknown |
| KM982946 | 2014 | CUHK | unknown |
| KM982947 | 2014 | CUHK | unknown |
| KM982948 | 2014 | CUHK | unknown |
| KM982949 | 2014 | CUHK | unknown |
| KM982950 | 2014 | CUHK | unknown |
| KM982951 | 2014 | CUHK | unknown |
| KM982952 | 2014 | CUHK | unknown |
| KM982953 | 2014 | CUHK | unknown |
| KM982956 | 2014 | CUHK | unknown |
| KP096327 | 2014 | CUHK | unknown |
| KP096328 | 2014 | CUHK | unknown |
| KP096329 | 2014 | CUHK | unknown |
| KP096330 | 2014 | CUHK | unknown |
| KP096331 | 2014 | CUHK | unknown |
| KP096332 | 2014 | CUHK | unknown |
| KP096334 | 2014 | CUHK | unknown |
| KP096335 | 2014 | CUHK | unknown |
| KP096336 | 2014 | CUHK | unknown |
| KP096337 | 2014 | CUHK | unknown |
| KP096340 | 2014 | CUHK | unknown |
| KP096341 | 2014 | CUHK | unknown |
| KP096342 | 2014 | CUHK | unknown |
| KP096343 | 2014 | CUHK | unknown |
| KP096344 | 2014 | CUHK | unknown |
| KP096345 | 2014 | CUHK | unknown |
| KP096346 | 2014 | CUHK | unknown |
| KP096348 | 2014 | CUHK | unknown |
| KP123606 | 2014 | CUHK | unknown |
| KP176393 | 2014 | CUHK | unknown |
| KP176394 | 2014 | CUHK | unknown |
| KP176395 | 2014 | CUHK | unknown |
| KP176396 | 2014 | CUHK | unknown |
| KP176397 | 2014 | CUHK | unknown |
| KP176399 | 2014 | CUHK | unknown |
| KP176400 | 2014 | CUHK | unknown |
| KP176401 | 2014 | CUHK | unknown |
| KP176402 | 2014 | CUHK | unknown |
| KP176403 | 2014 | CUHK | unknown |
| KP176404 | 2014 | CUHK | unknown |
| KP176405 | 2014 | CUHK | unknown |
| KP176406 | 2014 | CUHK | unknown |
| KP176407 | 2014 | CUHK | unknown |
| KP176408 | 2014 | CUHK | unknown |
| KP176409 | 2014 | CUHK | unknown |
| KP176410 | 2014 | CUHK | unknown |
| KP176411 | 2014 | CUHK | unknown |
| KP241905 | 2014 | CUHK | unknown |
| KP241906 | 2014 | CUHK | unknown |
| KP241907 | 2014 | CUHK | unknown |
| KP241908 | 2014 | CUHK | unknown |
| KP241909 | 2014 | CUHK | unknown |
| KP241910 | 2014 | CUHK | unknown |
| KP241912 | 2014 | CUHK | unknown |
| KP241913 | 2014 | CUHK | unknown |
| KP241914 | 2014 | CUHK | unknown |
| KP241915 | 2014 | CUHK | unknown |
| KP293587 | 2014 | CUHK | unknown |
| KP293588 | 2014 | CUHK | unknown |
| KP293589 | 2014 | CUHK | unknown |
| KP293590 | 2014 | CUHK | unknown |
| KP293591 | 2014 | CUHK | unknown |
| KP698923 | 2014 | CUHK | unknown |
| KP698924 | 2014 | CUHK | unknown |
| KP698925 | 2014 | CUHK | unknown |
| KP698926 | 2014 | CUHK | unknown |
| KP698927 | 2014 | CUHK | unknown |
| KP864105 | 2014 | CN | unknown |
| KP864106 | 2014 | CN | unknown |
| KP864107 | 2015 | CN | unknown |
| KR131783 | 2013 | IN | unknown |
| KR904221 | 2013 | ZA | unknown |
| KR904222 | 2013 | ZA | unknown |
| KR904223 | 2013 | ZA | unknown |
| KR904224 | 2013 | ZA | unknown |
| KR904226 | 2013 | ZA | unknown |
| KR904227 | 2013 | ZA | unknown |
| KR904237 | 2012 | ZA | unknown |
| KR904238 | 2013 | ZA | GII.Pe |
| KT202793 | 2013 | CN | GII.Pe |
| KT202794 | 2014 | CN | GII.Pe |
| KT202795 | 2014 | CN | GII.Pe |
| KT202796 | 2014 | CN | GII.Pe |
| KT202797 | 2014 | CN | GII.Pe |
| KT202798 | 2014 | CN | GII.Pe |
| KT780373 | 2015 | CUHK | unknown |
| KT780374 | 2015 | CUHK | unknown |
| KT780375 | 2015 | CUHK | unknown |
| KT780376 | 2015 | CUHK | unknown |
| KT780377 | 2015 | CUHK | unknown |
| KT780378 | 2015 | CUHK | unknown |
| KT780379 | 2015 | CUHK | unknown |
| KT780380 | 2015 | CUHK | unknown |
| KT780381 | 2015 | CUHK | unknown |
| KT780382 | 2015 | CUHK | unknown |
| KT780383 | 2015 | CUHK | unknown |
| KT780384 | 2015 | CUHK | unknown |
| KT780385 | 2015 | CUHK | unknown |
| KT780386 | 2015 | CUHK | unknown |
| KT780387 | 2015 | CUHK | unknown |
| KT780388 | 2015 | CUHK | unknown |
| KT780389 | 2015 | CUHK | unknown |
| KT780390 | 2015 | CUHK | unknown |
| KT780391 | 2015 | CUHK | unknown |
| KT780392 | 2015 | CUHK | unknown |
| KU311158 | 2014 | CAN | GII.Pe |
| KU678201 | 2015 | CNTW | GII.Pe |
| KU678202 | 2015 | CNTW | GII.Pe |
| KU678203 | 2016 | CNTW | GII.Pe |
| KU678204 | 2016 | CNTW | GII.Pe |
| KU678205 | 2016 | CNTW | GII.Pe |
| KU683739 | 2015 | CN | unknown |
| KU985152 | 2014 | GER | unknown |
| KU985153 | 2014 | GER | unknown |
| KU985154 | 2014 | GER | unknown |
| KU985155 | 2014 | GER | unknown |
| KU985156 | 2014 | GER | unknown |
| KU985157 | 2014 | GER | unknown |
| KU985159 | 2014 | GER | unknown |
| KU985160 | 2014 | GER | unknown |
| KU985161 | 2014 | GER | unknown |
| KU985162 | 2014 | GER | unknown |
| KU985164 | 2014 | GER | unknown |
| KX158283 | 2015 | CAN | GII.Pe |
| KX158285 | 2015 | CAN | GII.Pe |
| KX354004 | 2012 | US | unknown |
| KX354005 | 2012 | US | unknown |
| KX354009 | 2012 | US | unknown |
| KX354012 | 2012 | US | unknown |
| KX354014 | 2012 | US | unknown |
| KX354015 | 2012 | US | unknown |
| KX354017 | 2012 | US | unknown |
| KX354018 | 2012 | US | unknown |
| KX354019 | 2012 | US | unknown |
| KX354020 | 2012 | US | unknown |
| KX354021 | 2012 | US | unknown |
| KX354024 | 2012 | US | unknown |
| KX354025 | 2012 | US | unknown |
| KX354027 | 2012 | US | unknown |
| KX354028 | 2012 | US | unknown |
| KX354029 | 2012 | US | unknown |
| KX354030 | 2013 | US | unknown |
| KX354031 | 2013 | US | unknown |
| KX354032 | 2013 | US | unknown |
| KX354033 | 2013 | US | unknown |
| KX354034 | 2013 | US | unknown |
| KX354039 | 2013 | US | unknown |
| KX354040 | 2013 | US | unknown |
| KX354043 | 2013 | US | unknown |
| KX354044 | 2013 | US | unknown |
| KX354045 | 2013 | US | unknown |
| KX354048 | 2013 | US | unknown |
| KX354055 | 2013 | US | unknown |
| KX354056 | 2013 | US | unknown |
| KX354063 | 2013 | US | unknown |
| KX354064 | 2013 | US | unknown |
| KX354065 | 2013 | US | unknown |
| KX354066 | 2013 | US | unknown |
| KX354067 | 2013 | US | unknown |
| KX354068 | 2013 | US | unknown |
| KX354095 | 2014 | US | unknown |
| KX354096 | 2014 | US | unknown |
| KX354097 | 2014 | US | unknown |
| KX354098 | 2014 | US | unknown |
| KX354099 | 2014 | US | unknown |
| KX354100 | 2014 | US | unknown |
| KX354101 | 2014 | US | unknown |
| KX354102 | 2014 | US | unknown |
| KX354103 | 2014 | US | unknown |
| KX354104 | 2014 | US | unknown |
| KX354105 | 2014 | US | unknown |
| KX354106 | 2014 | US | unknown |
| KX354107 | 2014 | US | unknown |
| KX354108 | 2014 | US | unknown |
| KX354109 | 2014 | US | unknown |
| KX354110 | 2014 | US | unknown |
| KX354111 | 2013 | US | unknown |
| KX354112 | 2013 | US | unknown |
| KX354113 | 2014 | US | unknown |
| KX354114 | 2015 | US | unknown |
| KX354115 | 2015 | US | unknown |
| KX354116 | 2015 | US | unknown |
| KX354117 | 2015 | US | unknown |
| KX354120 | 2015 | US | unknown |
| KX354121 | 2015 | US | unknown |
| KX354122 | 2015 | US | unknown |
| KX354123 | 2015 | US | unknown |
| KX354129 | 2015 | US | unknown |
| KX354130 | 2015 | US | unknown |
| KX354138 | 2015 | US | unknown |
| KX354139 | 2016 | US | unknown |
| KX354140 | 2015 | US | unknown |
| KX354141 | 2015 | US | unknown |
| KX354142 | 2015 | US | unknown |
| KX354143 | 2015 | US | unknown |
| KX354144 | 2015 | US | unknown |
| KX354145 | 2015 | US | unknown |
| KX354146 | 2015 | US | unknown |
| KX371605 | 2013 | US | unknown |
| KX371606 | 2013 | US | unknown |
| KX371610 | 2013 | US | unknown |
| KX586330 | 2015 | CN | GII.Pe |
| KX657722 | 2016 | CNTW | GII.Pe |
| KX657723 | 2016 | CNTW | GII.Pe |
| KX657724 | 2016 | CNTW | GII.Pe |
| KX657725 | 2016 | CNTW | GII.Pe |
| KX657726 | 2016 | CNTW | GII.Pe |
| KX657727 | 2016 | CNTW | GII.Pe |
| KX657728 | 2016 | CNTW | GII.Pe |
| KX657729 | 2016 | CNTW | GII.Pe |
| KX657730 | 2016 | CNTW | GII.Pe |
| KX657731 | 2016 | CNTW | GII.Pe |
| KX657732 | 2016 | CNTW | GII.Pe |
| KX657733 | 2016 | CNTW | GII.Pe |
| KX657735 | 2016 | CNTW | GII.Pe |
| KX657736 | 2016 | CNTW | GII.Pe |
| KX657737 | 2016 | CNTW | GII.Pe |
| KX907727 | 2015 | US | GII.P16 |
| KX989469 | 2016 | CN | unknown |
| KX989470 | 2016 | CN | unknown |
| KX989471 | 2016 | CN | unknown |
| KX989472 | 2016 | CN | unknown |
| KX989473 | 2016 | CN | unknown |
| KY341923 | 2015 | HU | GII.Pe |
| KY406987 | 2015 | CN | unknown |
| KY406988 | 2015 | CN | unknown |
| KY406989 | 2015 | CN | unknown |
| KY406990 | 2015 | CN | unknown |
| KY406991 | 2015 | CN | unknown |
| KY406992 | 2015 | CN | unknown |
| KY406993 | 2015 | CN | unknown |
| KY406994 | 2015 | CN | unknown |
| KY406995 | 2015 | CN | unknown |
| KY406996 | 2015 | CN | unknown |
| KY406997 | 2015 | CN | unknown |
| KY406998 | 2015 | CN | unknown |
| KY406999 | 2015 | CN | unknown |
| KY407000 | 2015 | CN | unknown |
| KY407001 | 2015 | CN | unknown |
| KY407002 | 2015 | CN | unknown |
| KY407003 | 2015 | CN | unknown |
| KY407004 | 2015 | CN | unknown |
| KY407005 | 2015 | CN | unknown |
| KY407006 | 2015 | CN | unknown |
| KY407007 | 2015 | CN | unknown |
| KY407008 | 2015 | CN | unknown |
| KY407009 | 2015 | CN | unknown |
| KY407010 | 2015 | CN | unknown |
| KY407011 | 2015 | CN | unknown |
| KY407012 | 2015 | CN | unknown |
| KY407013 | 2015 | CN | unknown |
| KY407014 | 2015 | CN | unknown |
| KY407015 | 2015 | CN | unknown |
| KY407016 | 2015 | CN | unknown |
| KY407017 | 2015 | CN | unknown |
| KY407018 | 2015 | CN | unknown |
| KY407019 | 2015 | CN | unknown |
| KY407020 | 2015 | CN | unknown |
| KY407021 | 2015 | CN | unknown |
| KY407022 | 2015 | CN | unknown |
| KY407023 | 2015 | CN | unknown |
| KY407024 | 2015 | CN | unknown |
| KY407025 | 2015 | CN | unknown |
| KY407026 | 2015 | CN | unknown |
| KY407027 | 2015 | CN | unknown |
| KY407028 | 2015 | CN | unknown |
| KY407029 | 2015 | CN | unknown |
| KY407030 | 2015 | CN | unknown |
| KY407031 | 2015 | CN | unknown |
| KY407032 | 2015 | CN | unknown |
| KY407033 | 2015 | CN | unknown |
| KY407034 | 2015 | CN | unknown |
| KY407035 | 2015 | CN | unknown |
| KY407036 | 2015 | CN | unknown |
| KY407037 | 2015 | CN | unknown |
| KY407038 | 2015 | CN | unknown |
| KY407040 | 2015 | CN | unknown |
| KY407041 | 2015 | CN | unknown |
| KY407042 | 2015 | CN | unknown |
| KY407043 | 2015 | CN | unknown |
| KY407044 | 2015 | CN | unknown |
| KY407045 | 2015 | CN | unknown |
| KY407046 | 2015 | CN | unknown |
| KY407047 | 2015 | CN | unknown |
| KY407048 | 2015 | CN | unknown |
| KY407049 | 2015 | CN | unknown |
| KY407050 | 2015 | CN | unknown |
| KY407051 | 2015 | CN | unknown |
| KY407052 | 2015 | CN | unknown |
| KY407053 | 2015 | CN | unknown |
| KY407054 | 2015 | CN | unknown |
| KY407055 | 2015 | CN | unknown |
| KY407056 | 2015 | CN | unknown |
| KY407057 | 2015 | CN | unknown |
| KY407058 | 2015 | CN | unknown |
| KY407059 | 2015 | CN | unknown |
| KY407060 | 2016 | CN | unknown |
| KY407061 | 2016 | CN | unknown |
| KY407062 | 2016 | CN | unknown |
| KY407063 | 2016 | CN | unknown |
| KY407064 | 2016 | CN | unknown |
| KY407065 | 2016 | CN | unknown |
| KY407066 | 2016 | CN | unknown |
| KY407067 | 2016 | CN | unknown |
| KY407068 | 2016 | CN | unknown |
| KY407069 | 2016 | CN | unknown |
| KY407070 | 2016 | CN | unknown |
| KY407071 | 2016 | CN | unknown |
| KY407072 | 2016 | CN | unknown |
| KY407073 | 2016 | CN | unknown |
| KY407074 | 2016 | CN | unknown |
| KY407075 | 2016 | CN | unknown |
| KY407076 | 2016 | CN | unknown |
| KY407077 | 2016 | CN | unknown |
| KY407078 | 2016 | CN | unknown |
| KY407079 | 2016 | CN | unknown |
| KY407080 | 2016 | CN | unknown |
| KY407081 | 2016 | CN | unknown |
| KY407082 | 2016 | CN | unknown |
| KY407083 | 2016 | CN | unknown |
| KY407084 | 2016 | CN | unknown |
| KY407085 | 2016 | CN | unknown |
| KY407086 | 2016 | CN | unknown |
| KY407087 | 2016 | CN | unknown |
| KY407088 | 2016 | CN | unknown |
| KY407089 | 2016 | CN | unknown |
| KY407090 | 2016 | CN | unknown |
| KY407091 | 2016 | CN | unknown |
| KY407092 | 2016 | CN | unknown |
| KY407093 | 2016 | CN | unknown |
| KY407094 | 2016 | CN | unknown |
| KY407095 | 2016 | CN | unknown |
| KY407096 | 2016 | CN | unknown |
| KY407097 | 2016 | CN | unknown |
| KY407098 | 2016 | CN | unknown |
| KY407099 | 2016 | CN | unknown |
| KY407100 | 2016 | CN | unknown |
| KY407101 | 2016 | CN | unknown |
| KY407102 | 2016 | CN | unknown |
| KY407103 | 2016 | CN | unknown |
| KY407104 | 2016 | CN | unknown |
| KY407105 | 2016 | CN | unknown |
| KY407106 | 2016 | CN | unknown |
| KY407107 | 2016 | CN | unknown |
| KY407108 | 2016 | CN | unknown |
| KY407109 | 2016 | CN | unknown |
| KY407110 | 2016 | CN | unknown |
| KY407111 | 2016 | CN | unknown |
| KY407112 | 2016 | CN | unknown |
| KY407113 | 2016 | CN | unknown |
| KY407114 | 2016 | CN | unknown |
| KY407115 | 2016 | CN | unknown |
| KY407116 | 2016 | CN | unknown |
| KY407117 | 2016 | CN | unknown |
| KY407118 | 2016 | CN | unknown |
| KY407119 | 2016 | CN | unknown |
| KY407120 | 2016 | CN | unknown |
| KY407121 | 2016 | CN | unknown |
| KY407122 | 2016 | CN | unknown |
| KY407123 | 2016 | CN | unknown |
| KY407124 | 2016 | CN | unknown |
| KY407125 | 2016 | CN | unknown |
| KY407126 | 2016 | CN | unknown |
| KY407127 | 2016 | CN | unknown |
| KY407128 | 2016 | CN | unknown |
| KY407129 | 2016 | CN | unknown |
| KY407130 | 2016 | CN | unknown |
| KY407131 | 2016 | CN | unknown |
| KY407132 | 2016 | CN | unknown |
| KY407133 | 2016 | CN | unknown |
| KY407134 | 2016 | CN | unknown |
| KY407135 | 2016 | CN | unknown |
| KY407136 | 2016 | CN | unknown |
| KY407137 | 2016 | CN | unknown |
| KY407138 | 2016 | CN | unknown |
| KY407139 | 2016 | CN | unknown |
| KY407140 | 2016 | CN | unknown |
| KY407141 | 2016 | CN | unknown |
| KY407142 | 2016 | CN | unknown |
| KY407143 | 2016 | CN | unknown |
| KY407144 | 2016 | CN | unknown |
| KY407145 | 2016 | CN | unknown |
| KY407146 | 2016 | CN | unknown |
| KY407147 | 2016 | CN | unknown |
| KY407148 | 2016 | CN | unknown |
| KY407149 | 2016 | CN | unknown |
| KY407150 | 2016 | CN | unknown |
| KY407151 | 2016 | CN | unknown |
| KY407152 | 2016 | CN | unknown |
| KY407153 | 2016 | CN | unknown |
| KY407154 | 2016 | CN | unknown |
| KY407155 | 2016 | CN | unknown |
| KY407156 | 2016 | CN | unknown |
| KY407157 | 2016 | CN | unknown |
| KY407158 | 2016 | CN | unknown |
| KY407159 | 2016 | CN | unknown |
| KY407160 | 2016 | CN | unknown |
| KY407161 | 2016 | CN | unknown |
| KY407162 | 2016 | CN | unknown |
| KY407163 | 2016 | CN | unknown |
| KY407164 | 2016 | CN | unknown |
| KY407165 | 2016 | CN | unknown |
| KY407166 | 2016 | CN | unknown |
| KY407167 | 2016 | CN | unknown |
| KY407168 | 2016 | CN | unknown |
| KY407169 | 2016 | CN | unknown |
| KY407170 | 2016 | CN | unknown |
| KY407171 | 2016 | CN | unknown |
| KY407187 | 2016 | CN | unknown |
| KY407188 | 2016 | CN | unknown |
| KY407189 | 2016 | CN | unknown |
| KY407207 | 2016 | CN | unknown |
| KY407208 | 2016 | CN | unknown |
| KY407209 | 2016 | CN | unknown |
| KY407210 | 2016 | CN | unknown |
| KY407211 | 2016 | CN | unknown |
| KY407212 | 2016 | CN | unknown |
| KY421039 | 2015 | US | GII.Pe |
| KY488573 | 2014 | CNTW | GII.Pe |
| KY488574 | 2014 | CNTW | GII.Pe |
| KY488575 | 2015 | CNTW | GII.Pe |
| KY496327 | 2012 | US | GII.Pe |
| KY679160 | 2017 | CUHK | unknown |
| KY679161 | 2017 | CUHK | unknown |
| KY679162 | 2017 | CUHK | unknown |
| KY679163 | 2017 | CUHK | unknown |
| KY679164 | 2017 | CUHK | unknown |
| KY679165 | 2017 | CUHK | unknown |
| KY679166 | 2017 | CUHK | unknown |
| KY679167 | 2017 | CUHK | unknown |
| KY679168 | 2017 | CUHK | unknown |
| KY679169 | 2017 | CUHK | unknown |
| KY679170 | 2017 | CUHK | unknown |
| KY679171 | 2017 | CUHK | unknown |
| KY679172 | 2017 | CUHK | unknown |
| KY679173 | 2017 | CUHK | unknown |
| KY887599 | 2016 | UK | GII.P16 |
| KY887600 | 2016 | UK | GII.P16 |
| KY887601 | 2016 | UK | GII.P16 |
| KY887602 | 2015 | UK | GII.P16 |
| KY887603 | 2015 | UK | GII.P16 |
| KY887604 | 2015 | UK | GII.P16 |
| KY887605 | 2015 | UK | GII.P16 |
| KY905331 | 2016 | AU | GII.P4 |
| KY905333 | 2016 | AU | GII.Pe |
| KY905335 | 2016 | AU | GII.P16 |
| KY947549 | 2016 | US | GII.P16 |
| KY947550 | 2015 | US | GII.P16 |
| LC005725 | 2012 | JP | unknown |
| LC005726 | 2012 | JP | unknown |
| LC005732 | 2013 | JP | unknown |
| LC005735 | 2014 | JP | unknown |
| LC018706 | 2014 | JP | unknown |
| LC018707 | 2014 | JP | unknown |
| LC018708 | 2014 | JP | unknown |
| LC018709 | 2014 | JP | unknown |
| LC018710 | 2014 | JP | unknown |
| LC066046 | 2015 | JP | GII.Pe |
| LC101819 | 2012 | JP | unknown |
| LC153121 | 2016 | JP | GII.P16 |
| LC153122 | 2016 | JP | GII.P16 |
| LC175468 | 2016 | JP | GII.P16 |
| LC177655 | 2013 | VN | GII.Pe |
| LC177656 | 2013 | VN | GII.Pe |
| LC177657 | 2014 | VN | GII.Pe |
| LC177658 | 2015 | VN | GII.P4 |
| LC177659 | 2015 | VN | GII.Pe |
| LC177660 | 2015 | VN | GII.Pe |
| LC177661 | 2015 | VN | GII.Pe |
| LC325217 | 2016 | JP | GII.P16 |
| LC331997 | 2017 | JP | GII.P16 |
| LN854566 | 2014 | NL | GII.P4 |
| LN854567 | 2014 | NL | GII.Pe |
| MF140641 | 2013 | NL | GII.P4 |
| MF140642 | 2013 | NL | GII.P4 |
| MF140643 | 2013 | NL | GII.P4 |
| MF140644 | 2014 | NL | GII.P4 |
| MF140671 | 2013 | NL | GII.Pe |
| MF140672 | 2013 | NL | GII.Pe |
| MF140673 | 2013 | NL | GII.Pe |
| MF140674 | 2013 | NL | GII.Pe |
| MF140675 | 2014 | NL | GII.Pe |
| MF681695 | 2015 | BRA | unknown |
| MF681696 | 2016 | BRA | unknown |
| MG002630 | 2017 | AU | GII.P16 |
| MG002631 | 2017 | AU | GII.P16 |
| MG002633 | 2017 | AU | GII.P16 |
| MG214988 | 2017 | CN | GII.Pe |
